# The role of psychological insulin resistance in diabetes self‐care management

**DOI:** 10.1002/nop2.462

**Published:** 2020-02-19

**Authors:** Ancho Lim, Youngshin Song

**Affiliations:** ^1^ Chungnam National University College of Nursing Daejeon South Korea

**Keywords:** insulin therapy, older adults, self‐efficacy, self‐management, type 2 diabetes mellitus

## Abstract

**Aims:**

The purpose of this study was to identify the role of psychological insulin resistance in the relationship between diabetes self‐efficacy and diabetes self‐care management in people with diabetes over 65 years of age.

**Design:**

A descriptive, cross‐sectional design was used.

**Methods:**

Participants included 326 patients with type 2 diabetes who were over 65 years of age. Structural equation modelling was performed to estimate the direct and indirect effects of diabetes self‐efficacy on diabetes self‐care management when psychological insulin resistance was entered as a mediator. Data were collected from May 2015 to January 2017.

**Results:**

Diabetes self‐efficacy (*r *= .53, *p *< .001) and psychological insulin resistance (*r *= .33, *p *< .001) were significantly associated with diabetes self‐care management, whereas a negative association was found between diabetes self‐efficacy and psychological insulin resistance (*r *= −.16, *p *< .001). When psychological insulin resistance was entered as a mediator, the association between diabetes self‐efficacy and diabetes self‐care management was attenuated. Therefore, psychological insulin resistance served as a mediator of diabetes self‐care management.

## 1 INTRODUCTION

The global prevalence of diabetes was 9% as of 2017 (International Diabetes Foundation, 2017) and is expected to rise to 10% of the global population by 2040 (Ogurtsova et al., 2017). Approximately 23% of diagnosed patients are adults aged 65 and older (International Diabetes Foundation, 2017), and South Korea's prevalence among this group is especially high at 29.8% (Korean Diabetes Association, 2018). Diabetes is an incurable disease that requires lifelong maintenance of appropriate blood glucose levels to prevent further progression and complications (Kang & Gu, 2015). Patients with type 2 diabetes (T2D) are responsible for self‐care management (Ahola & Groop, 2013) that must be sustained over their lifetime (Kim et al., 2017). For older adults, in particular, increased life expectancy and medical advances have prolonged the amount of time people live with diabetes, making systematic blood glucose management ever more critical (Kim et al., 2007). Therefore, diabetes treatment for older adults should focus on minimizing the progression of diabetes, preventing further complications, maintaining optimal health (Bahrmann et al., 2014; Primožič, Tavčar, Avbelj, Dernovšek, & Oblak, 2012) and enhancing self‐care management along with medical intervention (Norris et al., 2002).

## 2 BACKGROUND

Diabetes self‐efficacy (DSE) is reported to be one of the most important predictors in effective self‐care management for diabetes (Abubakari, Cousins, Thomas, Sharma, & Naderali, 2016; Lee, Lee, & Moon, 2016; Song, Ahn, & Oh, 2013). DSE is a measure of the confidence of a person with diabetes and in his or her ability to care for him or herself and serves as an essential mechanism in diabetes self‐care (Rapley, Passmore, & Phillips, 2003). The American Association of Diabetes Educators (AADE) specifies DSE as one of the prerequisites for proper diabetes self‐care (American Diabetes Association, 2019). In other words, patients with higher DSE are better at self‐care management. Therefore, improving DSE is effective for regulating blood glucose levels and improving overall health (ADA, 2019; Al‐Khawaldeh, Al‐Hassan, & Froelicher, 2012; Liu et al., 2015; Rapley et al., 2003; Song et al., 2013).

Type 2 diabetes comprises 90% of diabetes in older adults. Insulin treatment is recommended for older persons with diabetes who have persistent hyperglycaemia despite taking oral hypoglycaemic agents, or have A1C 7.0% or greater after maximum or combined use of such agents (Korean Diabetes Association, 2013). Early insulin treatment has been recommended for older persons with T2D; however, older adults with T2D who refuse insulin therapy tend to have higher psychological insulin resistance than do those who have already received insulin (Bahrmann et al., 2014). Delayed insulin treatment caused by patients’ psychological insulin resistance (PIR) can lead to poor glycaemic control, complications and ultimately deteriorated quality of life (Nam, Chesla, Stotts, Kroon, & Janson, 2010). PIR can be attributed to psycho‐cognitive factors such as low awareness about insulin treatment and low confidence, as well as supportive factors, including the absence of diverse external support (Song, 2016). Hence, it is important to understand PIR and reduce it through education and counselling to promote proper self‐care management (Davis & Renda, 2006).

PIR is rooted in negative emotions associated with insulin treatment such as guilt, loss and failure (Brod, Kongso, Lessard, & Christensen, 2009) and is more prevalent in patients with low DSE (Brod et al., 2009; Larkin et al., 2008; Nam et al., 2010; Nam & Song, 2014). The results of the Survey for People who do not take Insulin (SPI), which was designed to identify the reasons why patients with diabetes refuse insulin treatment, showed a negative attitude towards insulin administration in subjects with poor self‐efficacy (Larkin et al., 2008). Self‐care education with an emphasis on DSE and communication with the healthcare provider must be conducted to reduce PIR (Nam et al., 2010). Healthy relationships with medical staff and proper DSE levels contribute to the lowering of PIR, so a PIR intervention programme must seek to improve DSE levels (Nam & Song, 2014).

Diabetes self‐care management (DSC) is integral to the maintenance of proper A1C levels and prevention of complications and is a decisive factor in patients’ quality of life (Song et al., 2013); it is also related to PIR (Bahrmann et al., 2014; Fu, Qiu, & Radican, 2009; Kuo et al., 2017; Polonsky et al., 1995). The Problem Area in Diabetes Survey (PAID), developed to measure psychosocial difficulties experienced by persons with diabetes, revealed that patients often experience psychosocial stress, which could hinder their DSC (Polonsky et al., 1995). PIR has several negative implications, including poor glycaemic control, increased risk of complications, deterioration of health, poor self‐care management and increased risk of death (Fu et al., 2009). The reduction of PIR in T2D patients leads to improved self‐care management and lower A1C levels (Kuo et al., 2017). Systematic evaluation of PIR and nursing strategies can effectively alleviate negative attitudes towards insulin and improve the quality of self‐care for patients (Bahrmann et al., 2014). Enhanced DSC through the reduction of PIR ensures the maintenance of proper A1C levels and leads to improved quality of life (Song et al., 2013). For older patients whose self‐care may be limited due to reduced physical activity and psychological, social and functional impairments caused by ageing, a nursing strategy that caters to their unique needs is required (Sohn & Yang, 2013). Therefore, it is crucial to understand the role PIR plays in the relationship between DSE and DSC.

## 3 HYPOTHESES OF THE STUDY

Based on the literature review, to confirm the role of PIR in the relationship between DSE and DSC, the authors proposed the following two hypotheses:

- (H1) both DSE and PIR would be associated with DSC.
- (H2) PIR would mediate the relationship between DSE and DSC.

## 4 METHOD

### 4.1 Design

This study used a cross‐sectional survey to examine the mediating role of PIR in the relationship between DSE and DSC. Structural equation modelling (SEM) was performed to estimate the direct and indirect effects of DSE on DSC when PIR was entered as a mediator.

### 4.2 Participants and procedures

A total of 326 patients with T2D were recruited from an outpatient clinic at a university hospital in D‐city and a public health centre in S‐city of South Korea. These locations were selected because the hospital was the largest in D‐city and had a large number of registered patients with diabetes. The public health care was the only health centre in S‐city and had a community diabetes management system. Data were collected using self‐report questionnaires, and details about the data collection protocol have been previously described (Song, Jeon, Cho, & Kim, 2016).

A sample size of more than 200 is recommended to estimate the fit the basic models in SEM (Kline, 2015). Eligible participants were 65 years of age or older, had A1C > 7.0 and were conscious and communicative. A1C was used for eligibility as it is the standard biomarker for glycaemic control and presents the average blood sugar for patients over the previous 2–3 months (ADA, 2019). The ADA (2019) guided that older adults with few comorbid chronic diseases and those who are cognitively intact should have a lower glycaemic goal.

In addition, they understood the purpose of the study and agreed to participate. Eligible participants with cognitive impairments, such as dementia, or difficulty answering the questions were excluded from the study.

### 4.3 Data collection and ethical consideration

This study used data collected from May 2015–January 2017 as part of the parent study, “Development of the Korean Psychological Insulin Resistance Measurement Tool and Evaluation of its Usability” (NRF‐2015RIA2A01002394). All research procedures were approved by the institutional review board at Chungnam National University (IRB No. 2‐104681‐A‐N‐01‐201705‐HR‐020‐09). Paper‐and‐pencil questionnaires were used for data collection. If participants had difficulty completing the questionnaire independently, they were completed by the participants with the help of a research assistant in a separate space. Written informed consent was obtained from each participant. The Strengthening the Reporting of Observational Studies in Epidemiology (STROBE), a 22‐item checklist, was used to confirm the quality of the cross‐sectional research conducted in this phase (von Elm et al., 2007; see File S1).

### 4.4 Measurement

#### 4.4.1 Psychological insulin resistance

PIR was measured using the Korean Psychological Insulin Resistance (K‐PIR) scale developed by Song (Song et al., 2016). The K‐PIR scale measures various aspects of psychological barriers to insulin treatment and attitudes towards insulin treatment in T2D patients. Responses are rated on a Likert scale ranging from 1 (*strongly disagree*)–5 (*strongly agree*) within two subscales: Psycho‐cognitive factors (14 items, including Negative Feelings, Low Awareness, Low Confidence for Self‐Injection, Dependency and Embarrassment) and Supportive factors (four items, including Economic Burden and Feelings About Supporters). Higher scores indicate a higher level of PIR. The K‐PIR questionnaire includes 18 items, and the range of possible scores is 18–90. Internal consistency as measured using the Cronbach's alpha reliability coefficient was 0.91 for this sample.

#### 4.4.2 Diabetes self‐efficacy

Diabetes self‐efficacy was measured using the Diabetes Self‐Efficacy Scale (DSES) developed by Rapley et al. (2003) and translated into Korean and tested for validity by Cho, Song, Jun, Lee, and Kim (2016). DSES is composed of five sub‐categories: diet (three items), self‐treatment (five items), routines (four items), certainty about one's self‐care (four items) and exercise (two items). The items of the survey are rated on a six‐point Likert scale from 1 (*not at all confident*)–6 (*totally confident*). Possible scores range from 18–108, with higher scores indicating higher DSE. Cronbach's alpha for this study was 0.87.

#### 4.4.3 Diabetes self‐care management

DSC was measured using the revised Summary of Diabetes Self‐care Activities (SDSCA) scale by Toobert, Hampson, and Glasgow (2000), which was translated into Korean by Lee, Park, and Park (2005) and tested for validity by Chang and Song (2009). The questionnaire consisted of a self‐report scale to assess how many days over the past 7 days, the participant engaged in self‐care as directed by the healthcare provider. The higher the score, the better the participant performed in his or her self‐care. The revised SDSCA scale consists of 11 items: Diabetes Diet (four items), Diabetes Exercise (two items), Self‐Monitoring of Blood Glucose (SMBG; two items), Diabetes Foot Care (two items) and Smoking (one item). The smoking item (i.e. whether the participant smokes cigarettes or not) was not included in this study. Cronbach's alpha for this study was 0.71.

### 4.5 Data analyses

In a mediating effect model, the independent variable (*X*) can exert a direct or indirect effect on the dependent variable (Baron & Kenny, 1986). If the relationship between the independent variable and the dependent variable is significantly influenced by the intervention of the mediating variable, it is called partial mediation. If it is not influenced significantly, it is referred to as full mediation (Hair, Black, Babin, & Anderson, 2006). In this study, the DSE was hypothesized to have an indirect effect via PIR on the DSC, and if it was associated with DSC without the mediation of PIR, it would have a direct association. In addition, partial mediation would occur when the relationship between DSE and DSC is significantly attenuated by PIR and full mediation would occur when the influence was not significant. SEM is a method of analysing research models based on hypotheses and can be used to confirm the relationship between latent variables and observed variables, as well as correlations and directionality among latent variables. Further, the confirmatory factor analysis model, regression model and complex path model can be verified using structural equation modelling (Hox & Bechger, 1998). Thus, SEM with a bootstrapping approach was used to test whether PIR mediated the relationship between DSE and DSC.

Path analysis and bootstrapping were used for descriptive statistics and to verify the SEM in this study, with statistical significance set at *p *< .05. Full Information Maximum Likelihood (FIML) estimation of the parameters was used to solve the missing data. FIML is considered to be a direct method as the model parameters and standard errors are estimated directly from the available data and no missing values are imputed (Yuan & Bentler, 2000).

Univariate skewness and kurtosis and multivariate kurtosis and critical ratio (CR) for the parameter estimates were checked to confirm the normality of the distribution. If univariate skewness and kurtosis were ranged from a value of −2–+2 and CR does not exceeds the critical value of |1.96| and |2.58|, it satisfied the normality of distribution for indicators (Kline, 2015). Data analysis was conducted using SPSS 22.0 and Analysis of Moment Structure (AMOS) software. General characteristics of the participants and measurement variables were analysed using descriptive statistics.

Hypothesis 1 The correlation between measurement variables was analysed using Pearson's Correlation Coefficient to confirm whether a correlation was established, which is a prerequisite for the analysis of the mediating effect.

Hypothesis 2 Path analysis and bootstrapping were performed to verify the mediating effect and significance of PIR, respectively. Chi‐squared goodness‐of‐fit (CMIN/DF, *χ* ^2^/*df*), standardized root mean residual (SRMR), normed fit index (NFI), comparative fit index (CFI), goodness‐of‐fit index (GFI) and root mean square error of approximation (RMSEA) were used to test the validity of the hypothetical model. Cut‐off values for model conformance were *χ* ^2^/*df* ≤ 3, SRMR ≤ 0.08, NFI ≥ 0.90, CFI ≥ 0.80, GFI ≥ 0.90 and RMSEA ≤ 0.10 (Hu, Bentler, & Kano, 1992).

## 5 RESULTS

### 5.1 Demographics

The characteristics of the participants (*N = *326) are presented in Table 1. The mean age of the participants was 73.2 (*SD* 5.7) years, and 52.1% were female. The mean HbA1C was 8.2% (*SD* 1.2, range 7.1–12.8). The mean duration of diabetes diagnosis was 13.8 years (*SD* 10.2). Most (70.6%) had never experienced insulin treatment management education. While insulin treatment was recommended for 121 (37.1%), only 88 (27%) were receiving it. The mean score of DSE, PIR and DSC was 70.7 (*SD* 15.4), 59.7 (*SD* 16.0) and 36.4 (*SD* 14.8), respectively.

**Table 1:** Demographics of participants

Characteristic	Categories or range	*N* (%) or Mean (±*SD*)
Age (years)	65–91	73.2 (±5.7)
A1C (%)	7.1–12.8	8.2 (±1.2)
Gender	Male	156 (47.9)
	Female	170 (52.1)
Has a spouse	Yes	237 (72.7)
	No	89 (27.3)
Education level	Non‐formal education	42 (12.9)
	Elementary school	109 (33.4)
	Middle school	60 (18.4)
	High school or higher	115 (35.2)
Has a job	Yes	67 (20.6)
	No	259 (79.4)
Perceived health status	1–5	3.2 (±0.8)
Length of diabetes (years)	0–50	13.8 (±10.2)
Number of comorbid conditions	0–8	0.9 (±1.2)
Insulin therapy	Yes	88 (27)
	No	238 (73)
Experienced insulin treatment management education	Yes	96 (29.4)
	No	230 (70.6)
Insulin therapy recommended	Yes	121 (37.1)
	No	205 (62.9)
Psychological insulin resistance	18–90	59.7 (± 16.0)
Diabetes self‐efficacy	18–108	70.7 (± 15.4)
Diabetes self‐care management	0–70	36.4 (± 14.8)

### 5.2 Hypothesis 1

The study's first hypothesis was that both DSE and PIR would be associated with DSC. All variables included in the study were statistically significant. DSC was negatively correlated with PIR (*r *= −.33, *p *< .001), but positively correlated with DSE (*r *= .53, *p *< .001). PIR was negatively correlated with DSE (*r *= −.16, *p *< .001). Therefore, the first prerequisite for mediation analysis was met for PIR, DSE and DSC.

### 5.3 Hypothesis 2

**Figure 1:**
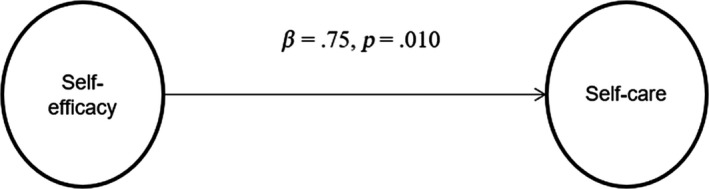
Direct effect of diabetes self‐efficacy on diabetes self‐care management

The second hypothesis tested was that PIR would mediate the relationship between DSE and DSC. The path model with the direct standardized coefficients is shown in Figure 1. The direct standardized effect of DSE on DSC was *β *= 0.75 (*p *= .010), indicating that participants with better DSE were directly associated with better DSC.

**Figure 2:**
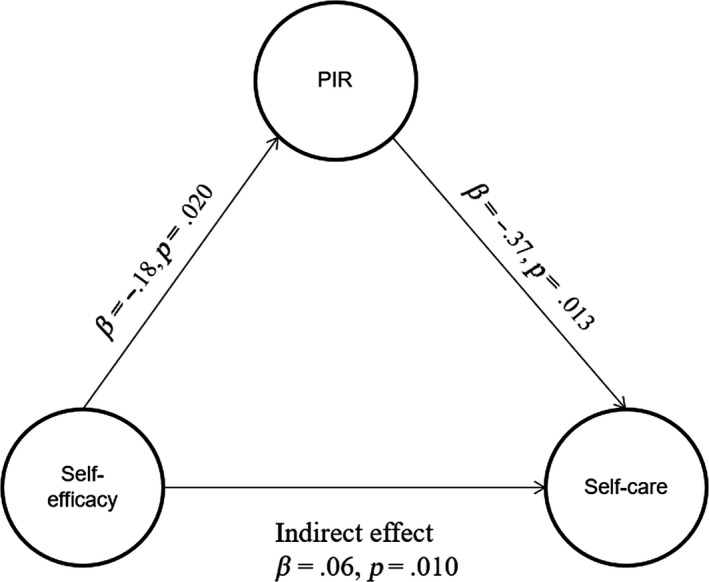
Partial mediation model of psychological insulin resistance in the relationship between diabetes self‐efficacy and diabetes self‐care management. PIR, psychological insulin resistance

An indirect effect between DSE and DSC was identified in the path diagram. As shown in Figure 2, the standardized indirect effect of DSE on DSC mediated by PIR was *β *= 0.06 (*p *= .010), indicating that better DSE that promotes DSC is in part due to less PIR. Participants who had greater DSE perceived less PIR (*β *= −0.18, *p *= .020) and less PIR indicated better DSC (*β *= −0.37, *p *= .013).

That is, a positive relationship between DSC and DSE in T2D patients remained significant after adjusting for PIR and there was a significant reduction in magnitude (*β* reduction in magnitude from 0.75–0.06) predicting the positive relationship between DSC and DSE (*β *= 0.06, *p *= .010); therefore, PIR partially mediated the prediction of diabetes self‐care by DSE. The indirect effect model fit indices included chi‐squared goodness‐of‐fit (*χ* ^2^/*df* = 6.464), SRMR < 0.0001, GFI* *= 0.873, NFI* *= 0.737, CFI* *= 0.765) and RMSEA* *= 0.130.

## 6 DISCUSSION

The findings from our study showed that better DSE was associated with greater DSC. In addition, PIR partially mediated the relationship between DSE and DSC in people over the age of 65.

### 6.1 Hypothesis 1

The major variables in this study, PIR, DSE and DSC, were found to have significant relationships with one another. DSE was positively correlated with DSC, and PIR had a negative correlation with DSE and DSC.

Diabetes self‐efficacy and DSC were positively correlated, which is consistent with the findings of previous studies (Abubakari et al., 2016; Lee et al., 2016; Song et al., 2013). DSE is the most influential factor in self‐care management. High DSE allows for stable maintenance of A1C levels by encouraging appropriate self‐care, ultimately improving the quality of a patient's life (Song et al., 2013). To improve DSC, nurses and healthcare providers must interact with patients, monitor their DSE and establish personalized goals and strategies to enhance DSE levels (Al‐Khawaldeh et al., 2012). For older patients, in particular, self‐care can be difficult due to psychological and physical changes brought on by ageing. Thus, a nursing strategy is needed that caters to their special needs and is different from the approach used with younger patients (Sohn & Yang, 2013). In this regard, the inclusion of self‐efficacy in individualized education and intervention programmes for older persons with diabetes would be meaningful for their sustainable self‐care management.

Diabetes self‐efficacy showed a negative correlation with PIR, meaning the higher one's level of DSE, the lower the PIR. This finding is consistent with another study involving patients who used oral hypoglycaemic agents, which demonstrated that patients with higher DSE levels had less resistance to insulin regardless of whether they were undergoing treatment due to their positive attitude towards insulin (Nam & Song, 2014). PIR was related to feelings of defeat, lack of command over one's life and low self‐efficacy (Larkin et al., 2008) and those with less self‐efficacy were found to have more PIR (Nam et al., 2010). Diagnosis of PIR and intervention in older patients enables early insulin treatment, which can improve their compliance as well as their quality of life (Kuo et al., 2017). The strategy that promotes self‐efficacy among older persons for the reduction of PIR seems highly necessary.

PIR was negatively correlated with diabetes self‐care, meaning that patients with low PIR perform better self‐care management. PIR can have negative consequences on a patient's health (Fu et al., 2009). Interventions to reduce PIR resulted in better self‐care management in elderly patients with T2D, resulting in decreased A1C levels (Kuo et al., 2017). Prior to the administration of insulin treatment for patients with T2D, proper mediation should take place to reduce their PIR; patients can perform satisfactory self‐care once they overcome PIR (Funnell, Kruger, & Spencer, 2004). With the duration of diabetes prevalence increasing, systematic blood glucose management and self‐care of older patients are growing in importance (Kim et al., 2007; Norris et al., 2002). Hence, incorporating a PIR reduction plan in insulin self‐injection training for T2D patients is suggested. A gradual, step‐by‐step approach is necessary to ensure that patients with T2D are supervised from the onset of the illness and assisted in overcoming their psychological aversion towards insulin.

### 6.2 Hypothesis 2

PIR was found to be a partially mediating variable in the relationship between DSC and DSE of persons with T2D.

Although the goodness‐of‐fit indexes did not meet all criteria, the research model can be accepted since both direct and indirect effects were shown to be statistically significant in our path analysis. This study has important implications in that it confirmed the role of PIR in the relationship between DSE and self‐care management, unlike previous studies that examined the relationships between self‐care management and DSE, DSE and PIR and PIR and self‐care separately. These findings suggest that PIR can play either a positive or negative role in the relationship between DSE and diabetes self‐care. Self‐care education curriculum with an emphasis on enhancing self‐efficacy, personal characteristics and communication with the healthcare provider is necessary to reduce PIR (Nam et al., 2010). PIR, in particular, is present in all patients diagnosed with T2D, regardless of whether insulin has been administered or not (Song, 2016), and should be treated through proper diabetes management, education and counselling (Davis & Renda, 2006). Therefore, it is critical to evaluate the degree of one's PIR from the diagnosis stage and make efforts to reduce it. Additionally, appropriately validated intervention and education programmes should be developed to reduce PIR, a mediating variable in the relationship between DSE and self‐care management of persons with diabetes.

There are several limitations to the current study. Although the research sample was large enough to test the hypothesis, it did not include diverse ethnic and racial groups. Convenience sampling used in this study may also hinder the accurate representation of patients with T2D. The inclusion of various racial and ethnics groups in the selection of the participants and subdividing them according to characteristics could be necessary.

Furthermore, variables showed low inter‐correlations. PIR relates exclusively to insulin while diabetes self‐care encompasses overall diabetes management, including diet, exercise and glycaemic control. Therefore, a detailed follow‐up study is proposed that explores whether enhanced DSE contributes to the reduction of PIR and whether reduced PIR promotes increased DSC. Despite such limits, this study confirmed the role of PIR in the relationship between DSE and DSC, providing important insight about necessary elements for enhancement of self‐care management for older patients and suggesting a direction for future academic approaches. Our research findings suggest integrating education and intervention for the reduction of PIR with self‐care management education. As shown by previous studies (Kim et al., 2015; Norris et al., 2002), personalized strategies tailored to the individual needs of patients with T2D will bring about positive changes to the patients' lives by improving self‐care management and result in reduction of medical costs for society as a whole.

## 7 CONCLUSIONS

The results suggested that diabetes self‐efficacy, as well as psychological insulin resistance, should be considered important factors for successful diabetes self‐care management. Our findings have clinical implications that are relevant for interventions designed to decrease PIR among individuals over 65 years old who have been diagnosed with T2D. Therefore, we suggest that DSE, as well as PIR, should be considered as important factors leading to successful DSC.

## 8 RELEVANCE TO CLINICAL PRACTICE

This study demonstrates that PIR is a mediator of the relationship between DSE and self‐care management. Thus, psychological insulin resistance in type 2 diabetes should be monitored consistently from the time of diagnosis and healthcare providers should implement appropriate tailored strategies for individual diabetes self‐care management. In future research, clinical nurses need to develop strategies to reduce the psychological insulin resistance of type 2 diabetes to improve self‐care management.

## CONFLICT OF INTEREST

There are no conflicts of interest.

## AUTHOR CONTRIBUTIONS

AH: data analysis and initial drafting of the paper. YS: design of the study and assistance in the data analysis, critical review and revision of the final draft.
